# Supplementary material for: Electrocatalytic Properties of Mixed-Oxide-Containing Composite-Supported Platinum for Polymer Electrolyte Membrane (PEM) Fuel Cells
Source: Materials (Basel). 2022 May 20;15(10):3671. doi: 10.3390/ma15103671 (PMC9148157; doi:10.3390/ma15103671)
Supplement: Supplementary file 1 [file materials-15-03671-s001.zip › materials-1714153-supplementary.pdf]

Supplementary Materials

Electrocatalytic Properties of Mixed-Oxide-Containing Composite-Supported Platinum for Polymer Electrolyte Membrane (PEM) Fuel Cells

Ilgar Ayyubov [1,2], Emília Tálas [1], Khirdakhanim Salmanzade [1,3], Andrei Kuncser [4], Zoltán Pászti [1], Ștefan Neațu [4], Anca G. Mirea [4], Mihaela Florea [4], András Tompos [1,*] and Irina Borbáth [1]

[1] Institute of Materials and Environmental Chemistry, Research Centre for Natural Sciences, Eötvös Loránd Research Network (ELKH), Magyar Tudósok körútja 2, H-1117 Budapest, Hungary; ayyubovi@gmail.com (I.A.); talas.emilia@ttk.hu (E.T.); ksalmanzade5@gmail.com (K.S.); paszti.zoltan@ttk.hu (Z.P.); borbath.irina@ttk.hu (I.B.)

[2] Department of Physical Chemistry and Materials Science, Faculty of Chemical Technology and Biotechnology, Budapest University of Technology and Economics, Műegyetem rkp. 3., H-1111 Budapest, Hungary

[3] Department of Inorganic and Analytical Chemistry, Faculty of Chemical Technology and Biotechnology, Budapest University of Technology and Economics, Műegyetem rkp. 3., H-1111 Budapest, Hungary

[4] National Institute of Materials Physics, 405A Atomistilor Street, 077125, Magurele, Romania; andrei.kuncser@infim.ro (A.K.); stefan.neatu@infim.ro (Ș.N.); anca.coman@infim.ro (A.G.M.); mihaela.florea@chimie.unibuc.ro (M.F.)

* Correspondence: tompos.andras@ttk.hu; Tel.: +36-1-382-501

Experimental

1. Preparation of TiO_2-based composite type of supports by multistep sol-gel method

The preparation of the mixed oxide – carbon composites (see Figure S1) consisted of three main steps: low temperature deposition of TiO_2-rutile nuclei on the carbon backbone completed by an aging step, introduction of the Mo precursor and incorporation of the Mo into the TiO_2-rutile crystallites using a high-temperature treatment step (HTT: Ar, 600 °C, 8 h) [1]. The main difference between the preparations of composite materials with low (25 weight% C) and high carbon content (75 weight% C) were (i) the duration and temperature of the aging step as well as (ii) second addition of the cc. HNO_3 before starting the aging procedure to compensate the whole acidity of the synthesis mixture. Upon the preparation of the composites with increased carbon content ($Ti_{0.8}Mo_{0.2}O_2$/C= 25/75) at the end of the 4-day aging procedure and before addition of Mo precursor compound the synthesis mixture was heated up to 65 °C for 8 h [1].

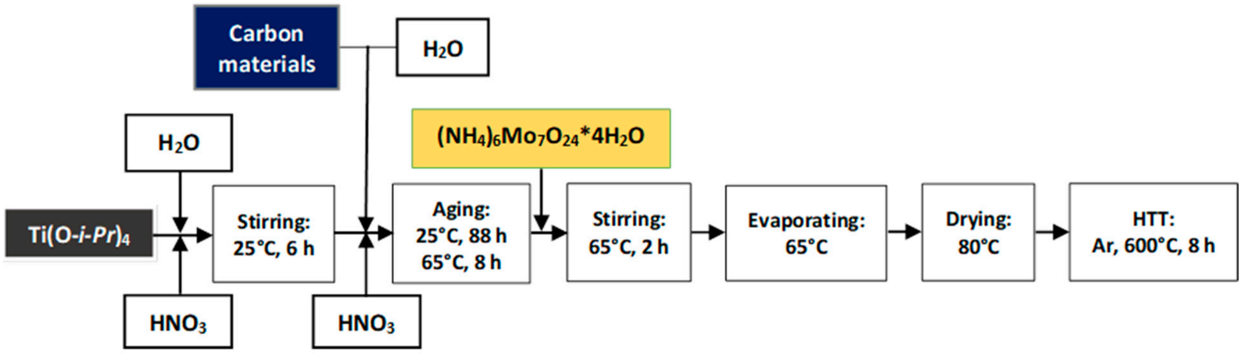

Figure S1. Flow chart for synthesis of 25 wt.% $Ti_{0.8}Mo_{0.2}O_2$-75 wt.% C type mixed oxide-carbon composites.

2. Preparation of the 20 wt.% Pt electrocatalysts

Composite support materials were loaded with 20 wt.% Pt via a modified $NaBH_4$-assisted ethylene-glycol (EG) reduction-precipitation method [2] which is demonstrated schematically in Figure S2.

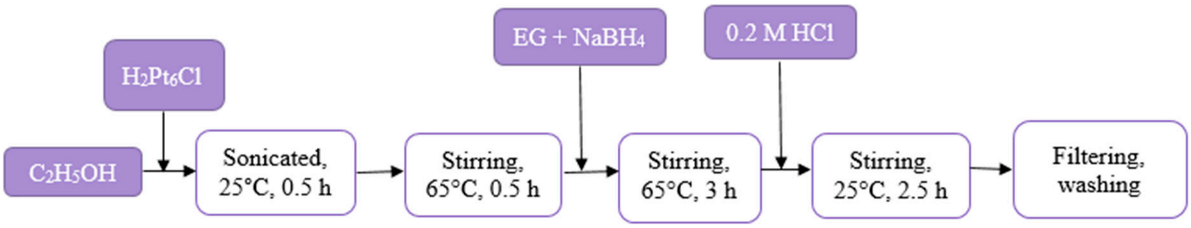

Figure S2. Flow chart for synthesis of 20 wt.% Pt electrocatalysts.

As a first step of the preparation procedure, $H_2PtCl_6 \times 6H_2O$ (134 mg) was dissolved in ethanol in a round bottom flask. The 200 mg of the support material was dispersed in the solution by sonication at room temperature and the suspension was heated up to 65 °C with continuous stirring. A solution prepared by mixing of $NaBH_4$ (596 mg) and EG (7.4 mL) was added dropwise to the suspension very slowly and carefully with a syringe pump in 30 min at 65 °C with continuous stirring. After the addition of $NaBH_4$ and EG in 30 min the system was stirred at 65 °C for 3 h. 0.2 M solution of hydrogen chloride (15 mL) was added to the suspension and it was stirred for an additional 2.5 h at room temperature in order to allow the Pt particles to settle on the surface of the support. The material was washed four times with 50 mL water and filtered by centrifugation in order to remove the chloride ions and dried at 85 °C in an oven overnight.

3. Electrochemical characterization of various batches of Pt electrocatalysts

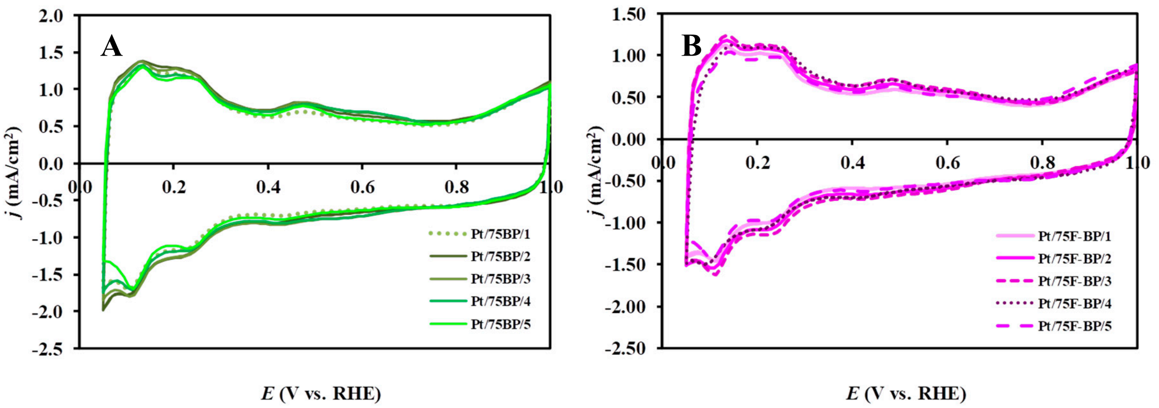

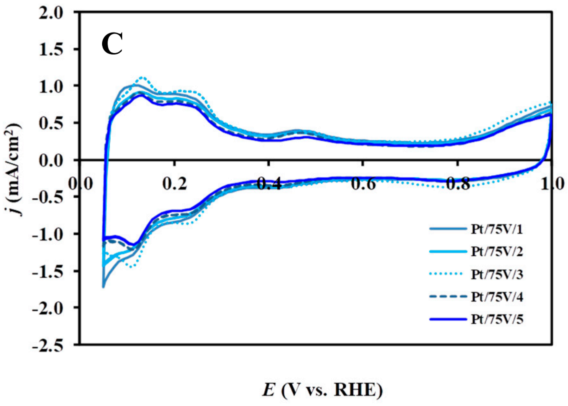

Figure S3. Electrochemical characterization of various batches of Pt electrocatalysts by cyclic voltammetry: (**A**) Pt/75BP, (**B**) Pt/75F-BP and Pt/75V (**C**). Cyclic voltammogram recorded in 0.5 M H_2SO_4 at 100 mV s^{-1}, T = 25 °C.

4. Physicochemical characterization of the composite supports and the electrocatalysts

XRD patterns were obtained in a Philips model PW 3710 based PW 1050 Bragg-Brentano parafocusing goniometer using CuKα radiation (λ= 0.15418 nm), graphite monochromator and proportional counter ((Philips, Eindhoven, Netherlands). Silicon powder (NIST SRM 640, National Institute of Standards and Technology, Gaithersburg, MD, USA) was used as an internal standard and the scans were evaluated with profile fitting methods. The cell parameters of the crystalline phases were determined from the fitted values. The average crystallite sizes of prepared composites were measured using Debye-Scherrer's equation.

Nitrogen physisorption measurements were carried out at temperature of liquid nitrogen using Thermo Scientific Surfer automatic volumetric adsorption analyzer (Thermo Fischer Scientific, Berlin, Germany). The specific surface area (S_{BET}) was calculated by the BET method in the range of relative pressures from 0.05 to 0.30.

Transmission Electron Microscopy (TEM) studies of the samples were made by use of a JEOL 3010 high resolution transmission electron microscope (Tokyo, Japan) operating at 300 kV. The structure has been identified by Selected Area Electron Diffraction (SAED) and the compositional homogeneity has been checked by Scanning Transmission Electron Microscopy (STEM) coupled with Energy Dispersive X-Ray

Spectroscopy (EDS). The samples for TEM analysis were prepared by dripping a few drops from the material suspension of the powder in ethanol on a microscopy grid with a carbon membrane.

Scanning electron micrographs of the samples were recorded with a scanning electron microscope Vega II LMU model from Tescan (Brno, Czech Republic), equipped with an energy dispersive X-ray spectrometer (EDX) Bruker Quantax 200 (Bruker Physik-AG, Karlsruhe, Germany), at the following operational parameters: accelerating voltage 30 kV, measuring time 1200 s, working distance around 17 mm, counting rate 0.4 kcps.

All Raman spectra were recorded on a LabRAM HR Evolution spectrometer from Horiba Jobin Yvon (HORIBA France SAS), with a laser radiation at wavelength of 633 nm. The spectra were recorded at room temperature in the extended scan mode in the 50–2000 cm^{-1} range.

For X-ray photoelectron spectroscopy (XPS) investigations either an EA 125 electron spectrometer manufactured by OMICRON Nanotechnology GmbH (Germany) with MgKα (1253.6 eV) excitation source and 1 eV spectral resolution or a PHI Quantera equipment (Physical Electronics, Inc, Chanhassen, MN, US) with monochromatized AlKα excitation (1486.6 eV) and a dual beam (electrons and Ar ions) charge neutralizer was used. The powdered composite supports and catalysts were suspended in isopropanol and drops of this suspension were dried onto stainless steel sample plates. Binding energies were referenced to the lowest binding energy contribution of the C 1s envelope, which was assigned to graphite-like (sp^2-hybridized) carbon in the active carbon backbone (284.4 eV).

The Pt content of the electrocatalysts was checked by inductively coupled plasma - optical emission spectrometry (ICP-OES) technique after microwave assisted dissolution in 1:8 mixtures of concentrated nitric acid and hydrofluoric acid. For the measurements a simultaneous SPECTRO GENESIS (SPECTRO Analytical Instruments GmbH, Kleve Germany) instrument with axial plasma observation was used.

5. Electrochemical characterization of composite supported electrocatalysts

All electrocatalysts were investigated by means of CV in a conventional three-electrode electrochemical glass cell using a Biologic SP150 potentiostat and the EC-LAB software package. The working electrode was prepared by supporting the electrocatalysts on a glassy carbon (GC) electrode (d= 0.3 cm, geometric surface area A= 0.0707 cm^2).

The GC electrode was preliminary polished by means of alumina powder (10 μm in diameter) with the addition of isopropanol in order to remove the impurities from the surface. Subsequently, the polishing alumina powder was removed from the electrode surface by rinsing with distilled water. The sample to be examined was pulverized and an ink was prepared by dispersing 2 mg of the catalyst sample in 2 mL of a mixture of 1.592 mL of MilliQ water, 0.4 mL of isopropanol and 8 μL of 5% Nafion® solution. The suspension was sonicated for 30 min. From this suspension a drop (3.6 μL) was pipetted on to the dry mirror-polished GC and dried at room temperature for 20 min.

The reference electrode was a hydrogen electrode immersed in the same electrolyte as the working electrode and all potentials are given on the reversible hydrogen electrode (RHE) scale. Pt was used as counter electrode. All electrochemical measurements were carried out at ambient temperature (25 °C). Solutions were prepared from Millipore MilliQ water; Ar 5.5 gas was used for deoxygenation of the solutions; the electrolyte was 0.5 M H_2SO_4.

Prior to the measurements, the electrode was activated by potential cycling for 10 times in the range 50 and 1000 mV at a scan rate of 100 mV s^{-1}. After the activation procedure, CV measurements were done in the potential range of 50–1000 mV at a scan rate of 100 mV s^{-1}.

The charges associated with hydrogen underpotential deposition, $Q_{ox_{Hupd}}$, were calculated from cyclic voltammograms. From the oxidation charge of the monolayer hydrogen the electrochemically active Pt surface area ($ECSA_{Hupd}$) can be calculated using the Equation (1) [3]:

$$ECSA_{Hupd}\ (cm^2) = Q_{ox_{Hupd}}\ (\mu C) / 210\ (\mu C/cm^2) \qquad (1)$$

where $ECSA_{Hupd}$ is the electrochemical surface area determined from the amount of underpotentially deposited hydrogen on the platinum surface; $Q_{ox_{Hupd}}$ is the oxidation charge of underpotentially deposited hydrogen obtained from the CV experiment and 210 (μC cm^{-2}) is the amount of charge required to oxidize one monolayer hydrogen adsorbed on 1 cm^2 of polycrystalline platinum surface.

Catalytic activity of the catalyst samples was tested in the ORR by rotating disc electrode (RDE) technique. A RDE is a glassy carbon working electrode used in a three-electrode system. The rotating speed of the electrode can be controlled, yielding variable diffusion rate of the reactant. The ORR measurements were done in O_2 saturated 0.5 M H_2SO_4 solution. The diameter of RDE electrode and Pt loading used in these experiments was the same as during CV measurements. Polarization curves were recorded by cathodic scan sweeping the potential between 1000 and 200 mV with 10 mVs^{-1} sweep rate, rotating the electrode at 225, 400, 625, 900, 1225 and 1600 revolutions/min (rpm).

Catalytic activity in the HOR was also investigated by RDE method in hydrogen saturated 0.5 M H_2SO_4 solution at 400, 625, 900, 1225 and 1600 rpm. Polarization curves were recorded by anodic scan sweeping the potential between 0 and 300 mV with 10 mVs^{-1} sweep rate. The diameter of RDE electrode and Pt loading used in these experiments was the same as during CV and ORR measurements.

In order to characterize the electrochemically active Pt surface area of the catalysts before and after the RDE measurements, 10 CVs between 50 and 1000 mV potential window in Ar saturated electrolyte were also measured.

In the long-term stability test, the samples were submitted to cyclic polarization at a 100 mVs^{-1} scan rate for 10,000 cycles between 50 and 1000 mV potential limits; these measurements took ca. 54 h.

For comparison, activity in the HOR and ORR and long-term stability of the commercially available reference Pt/C electrocatalyst with 20 wt.% Pt loading were also studied by the same methods as described above.

Results concerning on the change of the electrochemically active Pt surface area upon the 10,000-cycle stability test are presented as $ECSA_{10,000}$ normalized to $ECSA_1$ measured in the first cycle on the same sample. The measure of the ECSA loss after 10,000 cycles of the stability test is the $\Delta ECSA_{10,000}$ value defined in Equation (2) [1]:

$$\Delta ECSA_{10,000} = \{1-(ECSA_{10,000}/ECSA_1)\}\times 100\% \qquad (2)$$

Results

1. Results of physicochemical characterization

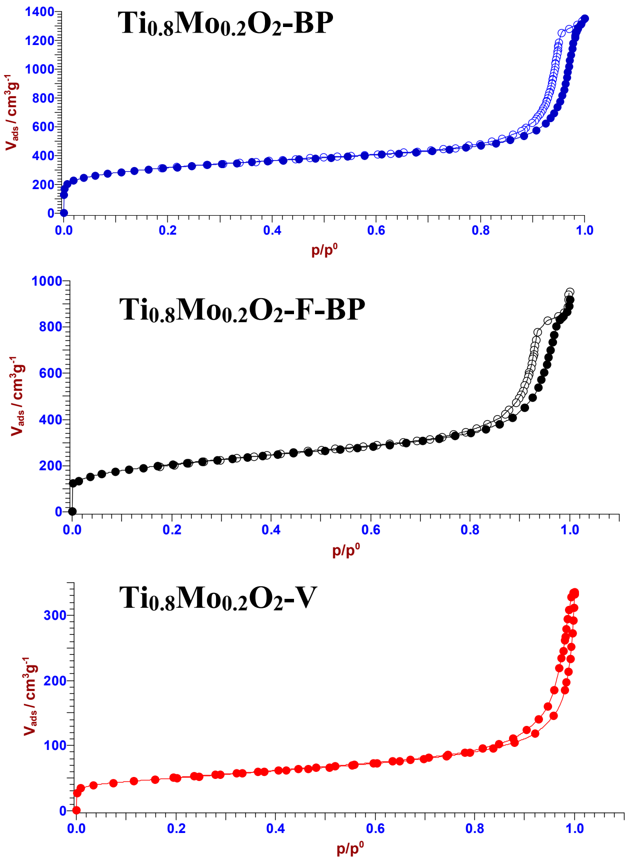

Figure S4. Nitrogen adsorption / desorption isotherms of the composite supports [1].

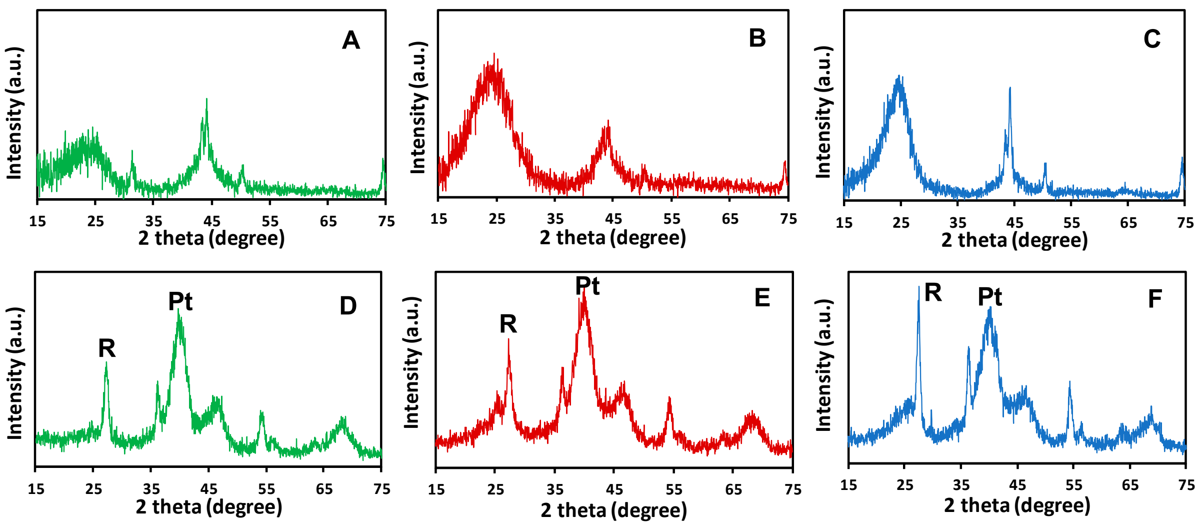

Figure S5. XRD patterns of the parent carbons and the composite supported Pt catalyst obtained from them. **A**: Black Pearls 2000 (BP), **B**: Functionalized Black Pearls 2000 (F-BP), **C** Vulcan XC-72 (V).

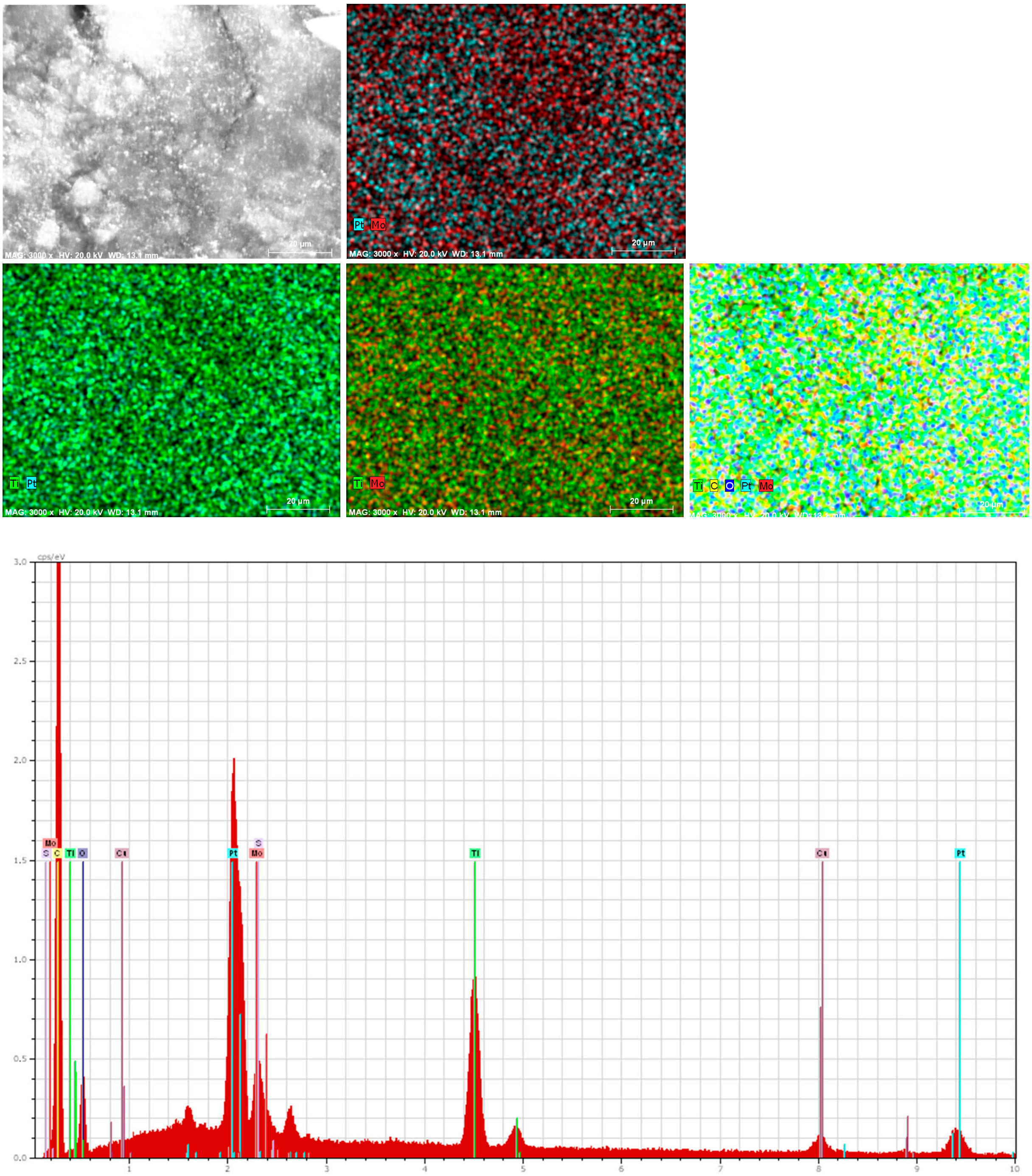

Figure S6. EDX elemental analysis of the Pt/75BP (20wt.% Pt/25 wt.% $Ti_{0.8}Mo_{0.2}O_2$-75 wt.% BP) electrocatalyst.

Table S1. Characterization of selected areas of the Pt/75BP sample by EDX.

Area	C (wt.%)	O (wt.%)	Ti (wt.%)	Mo (wt.%)	Pt (wt.%)
Pt-poor	72.5	12.8	8.1	3.5	3.1

Pt-rich	42.7	22.9	9.2	4.0	21.2

2. Results of electrochemical characterization

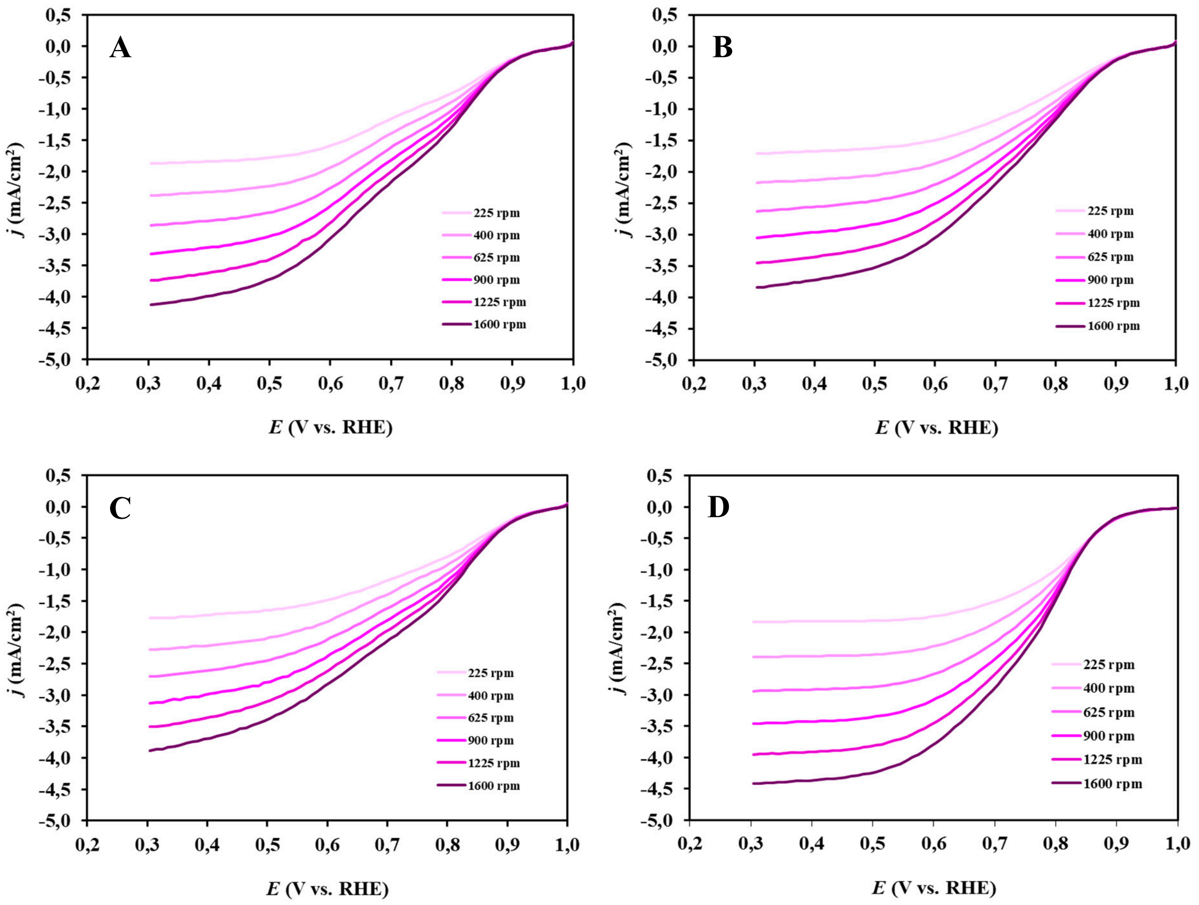

Figure S7. Potentiodynamic (10 mV s^{-1}, negative sweep) oxygen reduction current densities obtained in O_2-saturated 0.5 M H_2SO_4 at 225, 400, 625, 900, 1225 and 1600 rpm: (**A**) Pt/75BP, (**B**) Pt/75F-BP, (**C**) Pt/75V, and (**D**) Pt/C (QuinTech) electrocatalyst.

Table S2. The results of the Tafel analysis.

**Sample**	**ω (rpm)**	**Tafel slope (mV/decade) region I**	**j_0 (mA/cm^2) region I**	**Tafel slope (mV/decade) region II**	**j_0 (mA/cm^2) region II**	**α**
Pt/75BP	900	−61	1.84×10^{-6}	−121	4.23×10^{-4}	0.49
	1600	−61	1.84×10^{-6}	−116	3.46×10^{-4}	0.51
Pt/75F-BP	900	−54	4.11×10^{-7}	−120	3.94×10^{-4}	0.49
	1600	−56	5.76×10^{-7}	−116	3.24×10^{-4}	0.51
Pt/75V	900	−59	1.38×10^{-6}	−120	4.93×10^{-4}	0.49
	1600	−60	1.70×10^{-6}	−116	4.08×10^{-4}	0.51
Pt/C	900	−51	1.08×10^{-6}	−119	2.26×10^{-4}	0.50
	1600	−55	2.07×10^{-6}	−119	2.17×10^{-4}	0.50
	2025	−58	3.05×10^{-6}	−118	2.06×10^{-4}	0.50

j_0: exchange current density

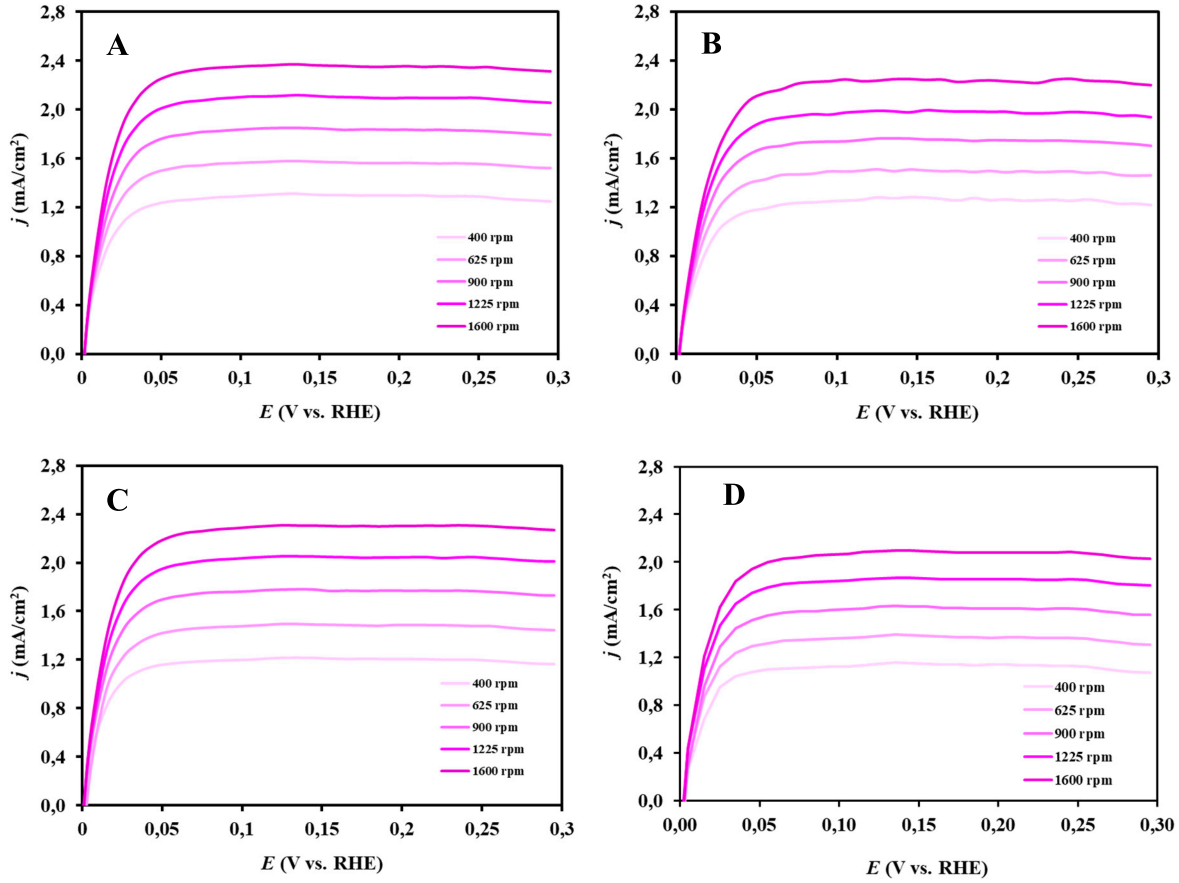

Figure S8. Potentiodynamic hydrogen oxidation current densities obtained in H_2-saturated 0.5 M H_2SO_4 at 400, 625, 900, 1225 and 1600 rpm: (**A**) Pt/75BP, (B) Pt/75F-BP, (**C**) Pt/75V, and (**D**) Pt/C (QuinTech) electrocatalyst. Sweep rate: 10 mV s^{-1}.

Table S3. Comparison of the literature data on the Koutecky - Levich (K-L) slope values obtained on different catalysts in the HOR.

**Catalyst**	**K-L slope,** **(mA/cm^2) rpm$^{-1/2}$**	**Reference**
20 wt.% Pt/Vulcan [b)]	5.72×10^{-2} (25 °C)<br>6.54×10^{-2} (theor) [d)]	T.J. Schmidt, H.A. Gasteiger, G.D. Stab, P.M. Urban, D.M. Kolb, R.J. Behm, Characterization of High-Surface-Area Electrocatalysts Using a Rotating Disk Electrode Configuration. J. Electrochem. Soc. 145 (1998) 2354–2358.
20 wt.% Pt/Vulcan (E-TEK) [a)]	~6.0×10^{-2} (25 °C)<br>6.0×10^{-2} (theor) [e)]	N. A. Maiorova, A. A. Mikhailova, O. A. Khazova, V. A. Grinberg, Thin-film Rotating Disk Electrode as a Tool for Comparing the Activity of Catalysts in the Hydrogen Oxidation Reaction. Russian Journal of Electrochemistry, 42(4) (2006) 331–338.
Pt-Ru bulk alloy [b)]	5.78×10^{-2} (25 °C)<br>6.54×10^{-2} (theor) [d)]	H.A. Gasteiger, N. Markovic, P.N. Ross, H_2 and CO Electrooxidation on Well-Characterized Pt, Ru, and Pt-Ru. 1. Rotating Disk Electrode Studies of the Pure Gases Including Temperature Effects. J. Phys. Chem., 99 (1995) 8290–8301.
20 wt.% Pt/Vulcan [a)]	7.77×10^{-2} (60 °C)	T.J. Schmidt, H.A. Gasteiger, R.J. Behm, Rotating Disk Electrode Measurements on the CO Tolerance of a High-Surface Area Pt/Vulcan Carbon Fuel Cell Catalyst. J. Electrochem. Soc. 146 (1999) 1296–1304.
Pt_3Sn alloy [b)]	8.1×10^{-2} (62 °C)	H.A. Gasteiger, N.M. Markovic, P.N. Ross, Electro-oxidation of CO and H_2/CO Mixtures on a Well-Characterized Pt_3Sn Electrode Surface. J. Phys. Chem. 99 (1995) 8945–8949.
20 wt.% $Pd_{0.8}Au_{0.2}$/Vulcan [a)]	8.0×10^{-2} (60 °C)	T.J. Schmidt, Z. Jusys, H.A. Gasteiger, R.J. Behm, U. Endruschat, H. Boennemann, On the CO tolerance of novel colloidal PdAu/carbon electrocatalysts. J. Electroanal. Chem. 501 (2001) 132–140.
5 wt.% Pt/$Ti_{0.7}W_{0.3}O_2$ [c)]	8.5×10^{-2} (25 °C) [f)]	D. Wang, C.V. Subban, H. Wang, E. Rus, F.J. DiSalvo, H.D. Abruña, Highly Stable and CO-Tolerant Pt/$Ti_{0.7}W_{0.3}O_2$ Electrocatalyst for Proton-Exchange Membrane Fuel Cells. J. Am. Chem. Soc. 132 (2010) 10218–10220.

[a)] stabilization of deposited catalyst particles on a glassy carbon RDE by coating with a thin film of Nafion;
[b)] UHV-prepared Pt-M alloy (M: Ru, Sn) electrodes mounted in a RDE configuration;
[c)] catalyst ink containing a mechanical mixture of a catalyst with carbon in Nafion solution, deposited on glassy carbon RDE;
[d)] the theoretical value of Bc_0 calculated at $c_0 = 7.14 \times 10^{-6}$ mol/cm^3 and $D = 3.7 \times 10^{-5}$ cm^2/s;
[e)] the theoretical value of Bc_0 calculated at $c_0 = 9.7 \times 10^{-7}$ mol/cm^3 and $D = 2.05 \times 10^{-5}$ cm^2/s;
[f)] electrolyte: 0.1 M H_2SO_4.

References

1. Borbáth, I.; Tálas, E.; Pászti, Z.; Zelenka, K.; Ayyubov, I.; Salmanzade, K.; Sajó, I.E.; Sáfrán, G.; Tompos, A. Investigation of Ti-Mo Mixed Oxide-Carbon Composite Supported Pt Electrocatalysts: Effect of the Type of Carbonaceous Materials. *Appl. Catal. A Gen.* **2021**, *620*, 118155.
2. Vass, Á.; Borbáth, I.; Pászti, Z.; Bakos, I.; Sajó, I.E.; Németh, P.; Tompos, A. Effect of Mo Incorporation on the Electrocatalytic Performance of Ti–Mo Mixed Oxide–Carbon Composite Supported Pt Electrocatalysts. *React. Kinet. Mech. Catal.* **2017**, *121*, 141–160
3. Woods, R. *Electroanalytical Chemistry: A Series of Advances*; Bard, A.J., Ed.; Marcel Dekker Inc.: New York, NY, USA; Basel, Switzerland, 1976; Volume 9, pp. 1–162.
